# Lung ultrasound to evaluate pulmonary changes in patients with cardiogenic shock undergoing extracorporeal membrane oxygenation: a retrospective study

**DOI:** 10.1186/s12871-023-02134-9

**Published:** 2023-05-25

**Authors:** Rongguo Wang, Meiyan Zhou, Yuanyuan Man, Yangzi Zhu, Wenping Ding, Qian Liu, Bin Sun, Li Yan, Yan Zhang, Hai Zhou, Liwei Wang

**Affiliations:** 1 Department of Anesthesiology, Xuzhou Central Hospital, Xuzhou, China; 2 Department of Respiratory, Xuzhou Central Hospital, Xuzhou, China

**Keywords:** Venous-arterial extracorporeal membrane oxygenation, Lung ultrasound score, Cardiogenic shock

## Abstract

**Purpose:**

The aim of the study was to evaluate the value of lung ultrasound (LUS) in patients with cardiogenic shock treated by venoarterial extracorporeal membrane oxygenation (VA-ECMO).

**Methods:**

A retrospective study was conducted in Xuzhou Central Hospital from September 2015 to April 2022. Patients with cardiogenic shock who received VA-ECMO treatment were enrolled in this study. The LUS score was obtained at the different time points of ECMO.

**Results:**

Twenty-two patients were divided into a survival group (n = 16) and a nonsurvival group (n = 6). The intensive care unit (ICU) mortality was 27.3% (6/22). The LUS scores in the nonsurvival group were significantly higher than those in the survival group after 72 h (P < 0.05). There was a significant negative correlation between LUS scores and PaO_2_/FiO_2_ and LUS scores and pulmonary dynamic compliance(Cdyn) after 72 h of ECMO treatment (*P* < 0.001). ROC curve analysis showed that the area under the ROC curve (AUC) of T_72_-LUS was 0.964 (95% CI 0.887 ~ 1.000, P < 0.01).

**Conclusion:**

LUS is a promising tool for evaluating pulmonary changes in patients with cardiogenic shock undergoing VA-ECMO.

**Trial registration:**

The study had been registered in the Chinese Clinical Trial Registry(NO.ChiCTR2200062130 and 24/07/2022).

## Introduction

Even with important advances in revascularization strategies and heart failure pharmacotherapies, venoarterial extracorporeal membrane oxygenation (VA-ECMO) has been widely used to provide life support in patients with refractory cardiogenic shock and has significantly improved their survival rate [1, 2]. Although VA-ECMO can significantly improve organ perfusion and oxygenation in cardiogenic shock patients, it can cause pulmonary oedema (PE), a common complication in at least 20–30% of patients [3], and pulmonary dysfunction similar to acute respiratory distress syndrome (ARDS) [4].

Lung computed tomography (CT) is regarded as the gold standard for noninvasive evaluation of pulmonary oedema, but it is not convenient for critically ill patients with cardiogenic shock undergoing VA-ECMO due to the exposure to ionizing radiation and the need for transportation, which limits its application [5]. At present, lung ultrasound has been widely used to evaluate the condition of severe patients. The lung ultrasound (LUS) has been proven to be an alternative method for monitoring pulmonary oedema and accurately assessing the severity of ARDS [6, 7]. LUS has been used to assess lung changes in patients with COVID-19 in intensive care units who were treated with ECMO, and the LUS results have been compared with C-reactive protein (CRP) and ventilator settings [8].

However, the application of bedside LUS in patients with cardiogenic shock undergoing VA-ECMO support has rarely been reported and has not been systematically studied. In this study, patients with cardiogenic shock who received VA-ECMO were reprospectively enrolled, and the LUS score was obtained before and during VA-ECMO support. The aim of this study was to assess the potential utility of the LUS score in patients receiving VA-ECMO support.

## Materials and methods

### Study population

A retrospective study was conducted in a tertiary-level teaching hospital (Xuzhou Central Hospital). The study protocol was reviewed and approved by the Ethics Committee of our hospital and conforms to the Declaration of Helsinki.

Between September 2015 and April 2022, twenty-two patients who underwent VA-ECMO support and LUS measurement and with cardiogenic shock were enrolled in the study. Eleven patients had acute myocardial infarction, 8 had fulminant myocarditis, and 3 had heart failure after cardiac surgery. The patients were divided into survivors (n = 16) and nonsurvivors (n = 6).

In our centre, when the following criteria are met, VA-ECMO treatment is required for patients with refractory cardiogenic shock [9]: (1) Persistence or aggravation of tissue hypoxia (extensive skin mottling, anuria, neurological impairment, elevated blood lactate, etc.) despite adequate fluid loading; or (2) sustained hypotension (systolic blood pressure < 90mmHg or mean arterial pressure < 65 mmHg) despite infusion of very-high-dose catecholamines (epinephrine ≥ 0.3 µg/kg/min, dopamine ≥ 15 µg/kg/min, norepinephrine ≥ 0.3 µg/kg/min). The exclusion criteria included the following: (1) ECMO duration less than 3 days; (2) lack of an appropriate acoustic window for LUS determination; (3) complications with pneumothorax; (4) complications with congenital heart disease; or (5) complications with chronic lung disease.

### Data Collection

Demographic and clinical data were collected from the hospital records and our organization’s proprietary database. The demographic data included age, sex, and body mass index (BMI). The clinical data included the Acute Physiology and Chronic Health Evaluation II (APACHE II) score, comorbidities, ICU mortality, length of ICU stay, duration of ECMO and duration of ventilation.

### Extracorporeal membrane oxygenation

The ECMO setup involved femoral vein catheterization, femoral arterial catheterization and superficial femoral artery catheterization [10]. The femoral arterial cannula (16-18 F for adults, Medtronic) was inserted into the common femoral artery and positioned in the distal aorta. A vein cannula (20-24 F for adults, Medtronic) was inserted into the femoral vein and positioned in the inferior vena cava close to the right atrium. A 7 F catheter was inserted into the superficial artery to prevent leg ischaemia [11]. The blood flow of VA-ECMO was controlled at 80-100ml/kg. The activated clotting time was maintained at 160–220s. An intraaortic balloon pump (IABP) was applied for all patients to maintain haemodynamic stability.

The adult-related indications for ECMO weaning were used as a reference in our centre, which included the following [12]: stable haemodynamic conditions ≥ 2–4 h; dosages of vasoactive drugs such as dopamine and dobutamine < 10 µg/kg/min; oxygen saturation of internal jugular vein > 70%; pulse pressure close to normal; LV ejection fraction (LVEF) > 40%; central venous pressure ≤ 12 mmHg.

### Mechanical ventilation

A protective mechanical ventilation strategy was implemented during ECMO for all the patients, and standardized nursing work was performed under the guidance of doctors. The protective mechanical ventilation strategy included [13–15] selection of the pressure control (PC) mode and limiting the tidal volume to < 4 ml/kg, the respiratory rate to 6 ~ 20 times/min, the inspiratory peak pressure to 20 ~ 25 cmH_2_O, the positive end expiratory pressure (PEEP) between 10 ~ 15 cmH_2_O, and the oxygen intake concentration between 30 − 50%, and the setting of respiratory rate refers to the changes of tidal volume and ECMO airflow to match. The pH value and arterial blood carbon dioxide partial pressure measured by arterial blood gas analysis were maintained within the normal range.

### Lung ultrasound score

Based on the scheme proposed by Bouhemad et al [16], the patients’ chests were divided into 12 regions. Each hemithorax is systematically divided into six regions, two anterior, two lateral, and two posterior, according to the anatomical landmarks set by the anterior and posterior axillary lines. Each region is divided into half, superior, and inferior. To perform a comprehensive examination, all adjacent intercostal spaces were explored in each region of interest by sliding the probe along the space. For each explored region, the most severe finding was reported in simple checkboxes according to the following rating: normal: 0; well-separated B-lines: 1; coalescent B-lines: 2; and consolidation: 3. The cumulative lung ultrasound (LUS) score corresponds to the sum of each examined region score (minimum score, normal lungs: 0; maximum score, both consolidated lungs: 36). ANT = anterior; INF = inferior; LAT = lateral; POST = posterior; SUP = superior. All procedures were performed by trained sonographers. For a full inspection with a 13 − 6 MHz linear probe (M-Turbo portable colour ultrasound, Sono), all the adjacent intercostal spaces of each region were explored.

The lung ultrasound scores of the patients were obtained at different periods, including at the initiation of ECMO, each morning in the following four days and at the termination of ECMO and ventilation as T_0_, T_24_, T_48_, T_72_, T_96_, T_W_ and T_R_. According to the above periods, the corresponding ventilator parameters and the blood gas analysis results of the right upper limb artery blood sample were also recorded, including PaO_2_/FiO_2_, PaCO_2_, and pulmonary dynamic compliance (Cdyn).

### Statistical analysis

Statistical analysis was performed with IBM SPSS Statistics 26 (IBM Corp., Armonk, NY). Normal distribution was formally tested with the Shapiro–Wilk test. Continuous data are presented as the mean with standard deviation. ANOVA was used to compare repeated measurement data at different time points, and a T test was used for comparisons between two groups. Categorical data are presented as frequencies and percentages. Categorical variables were compared using the chi-square test. The Pearson method was used to compare the correlation between the LUS score and PaO_2_/FiO_2_. ROC curves analysis was used to determine the diagnostic value of the significantly changed variables during ECMO support for the ICU survival status of the patients. A *p* value < 0.05 was considered statistically significant.

## Results

### Patient characteristics

Twenty-two patients with refractory cardiogenic shock who received VA-ECMO support in our centre were included in the study, including 13 males and 9 females aged 14 to 77 years. There were 11 cases of acute myocardial infarction, 8 cases of fulminant myocarditis and 3 cases of cardiac postoperative assistance. All patients received endotracheal intubation and ventilator-assisted breathing.

According to discharge survival status, the patients were divided into a survival group (n = 16) and a nonsurvivor group (n = 6). There were no significant differences with regard to age, sex, basic diseases, APACHE II, ECMO running time, or mechanical ventilation support time between the two groups (all *P* > 0.05). However, the length of ICU stay in the survival group was longer than that in the nonsurvivor group (*P* < 0.05). Detailed baseline characteristics for the study population are shown in Table 1.

**Table 1:** Baseline characteristics of the patients at the initiation of ECMO support

Characteristics	Survival group(n = 16)	Nonsurvival group(n = 6)	*χ*^2^/*t-* value	*P value*
Age, year	51.75±16.03	38.67±20.07	1.596	0.4581
Male, *n*(%)	12(75.0)	5(83.3)	0.415	0.678
BMI, kg/m2	24.54±1.87	24.69±2.26	0.154	0.879
APACHE II	25.31±4.56	23.33±3.21	0.971	0.343
**Comorbidity**				
hypertension, *n*(%)	4(25.0)	1(16.7)	0.415	0.678
Diabetes, *n*(%)	3(18.6)	1(16.7)	0.113	0.910
COPD, *n*(%)	2(12.5)	1(16.7)	0.254	0.799
ECMO running time, h	133.9±12.65	178.5±45.95	0.063	2.346
Mechanical ventilation support time, h	170.5±16.84	191.7±43.05	0.105	1.701
Length of stay in ICU, h	235.0±22.49	195.7±46.10	2.723	0.013*

LUS, lung ultrasound; ECMO, extracorporeal membrane oxygenation; Cdyn, dynamic lung compliance

### Lung ultrasound score and respiratory mechanics

**Fig. 1:**
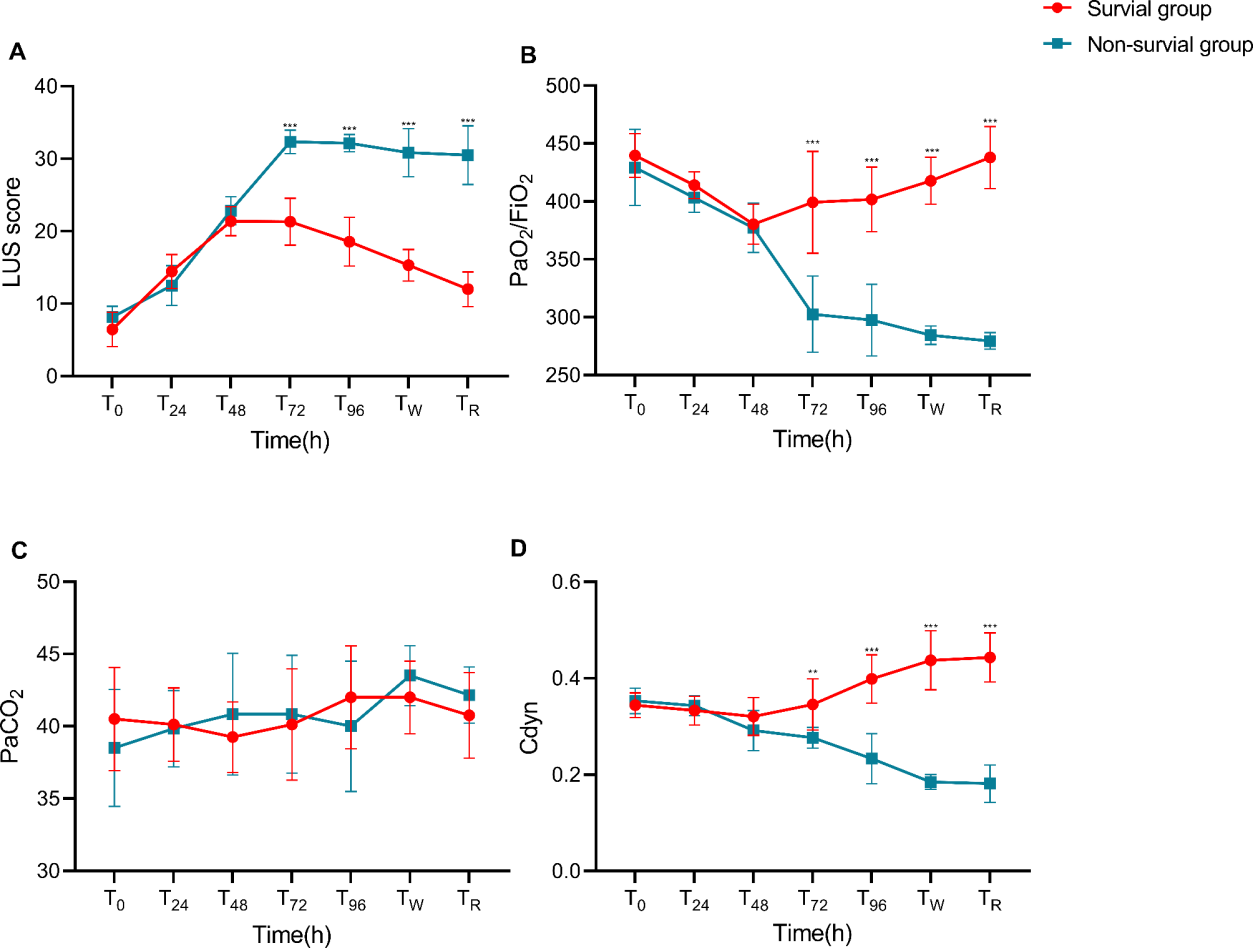
Comparison of the LUS score (**A**), PaO2/FiO2 (**B**), PaCO2 (**C**) and respiratory compliance (**D**) during ECMO between the survivors and nonsurvivors

Data The lung ultrasound score in the two groups was lower than T_0_ on T_24_, T_48_, T_72_, T_96_, T_W_ and T_R_ (*P* < 0.05). The LUS scores at T_72_, T_96_, T_W_ and T_R_ in the nonsurvivor group were higher than those in the survival group, and the differences were statistically significant (*P* < 0.05). Repeated measures analysis of variance was calculated as follows: F_Time_ = 165.7 (*P* < 0.001); F_Group_ = 142.6 (*P* < 0.001); F_Time × grouping_ = 54.30 (*P* < 0.001) (Fig. 1A).

The PaO_2_/FiO_2_ ratio in the survival group was lower than that in the T_0_ group at T_24_, T_48_, T_72_ and T_96_ (*P* < 0.05). PaO_2_/FiO_2_ in the nonsurvivor group was lower than that at T_0_ at T_48_, T_72_, T_96_, T_W_ and T_R_ (*P* < 0.05). PaO_2_/FiO_2_ at T_72_, T_96_, T_W_ and T_R_ in the nonsurvivor group was lower than that in the survival group, and the difference was statistically significant (*P* < 0.05). Repeated measures analysis of variance was as follows: F_Time_ = 30.96 (*P* < 0.001); F_Group_ = 262.9 (*P* < 0.001); F_Time × grouping_ = 28.92 (*P* < 0.001) (Fig. 1B).

PaCO2 analysis showed that there was no significant difference in time, intergroup differences or interaction between the two groups (*P* > 0.05). Repeated measures analysis of variance was as follows: F_Time_ = 2.464 (*P* < 0.05); F_Group_ = 0.025 (*P* > 0.05); F_Time × grouping_ =1.286 (*P* > 0.05) (Fig. 1C).

Cdyn in the survival group was lower than that at T_0_ compared to at T_96_, T_W_ and T_R_ (*P* < 0.05). Cdyn in the nonsurvivor group was lower than that at T_0_ at T_2_4, T_48_, T_72_, T_96_, T_W_ and T_R_ (*P* < 0.05). Cdyn at T_72_, T_96_, T_W_ and T_R_ in the nonsurvivor group was lower than that in the survival group, and the difference was statistically significant (*P* < 0.05). Repeated measures analysis of variance was as follows: F_Time_ = 3.000 (*P* < 0.05); F_Group_ = 94.13 (*P* < 0.001); F_Time × grouping_ = 39.40 (*P* < 0.001) (Fig. 1D).

### Correlation of LUS score and PaO2/FiO2

**Fig. 2:**
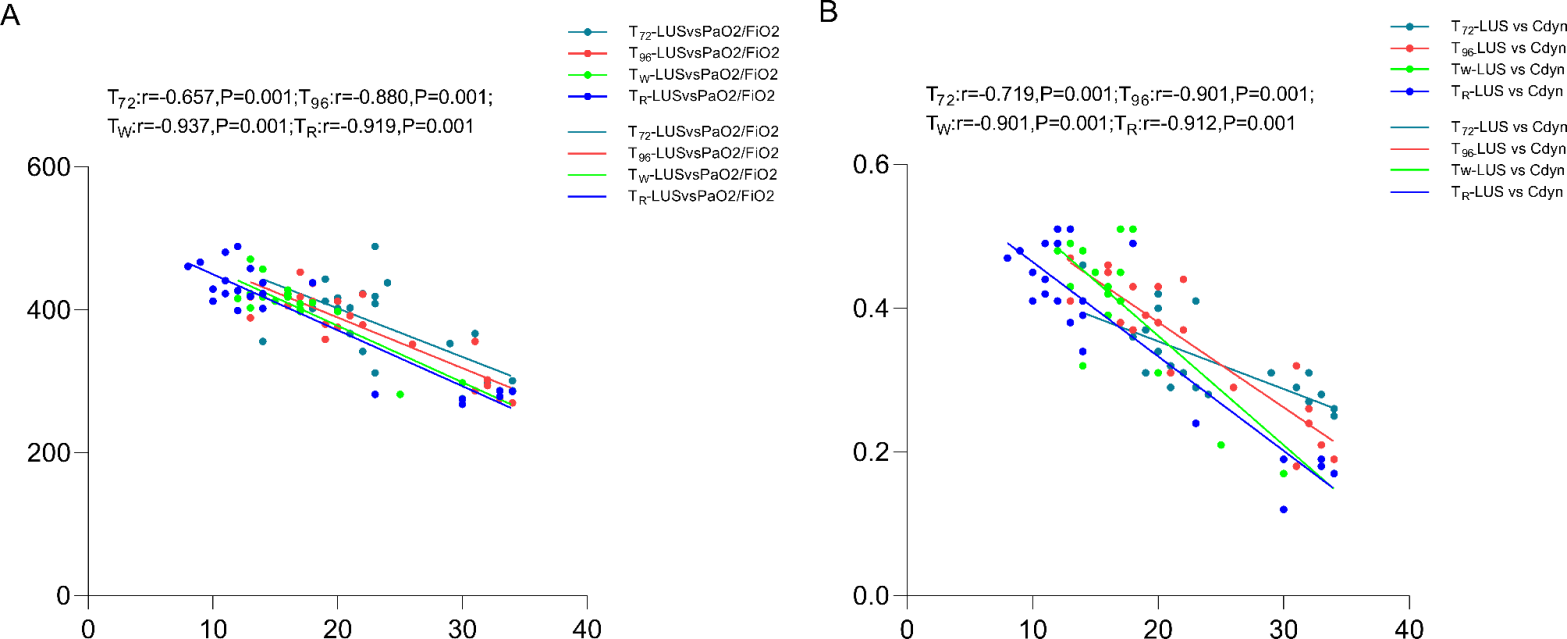
Scatterplots demonstrating the correlation between the LUS score and PaO2/FiO2 (**A**) and Cdyn (**B**)

The correlation analysis showed that there was a significant negative correlation between the lung ultrasound score and PaO2/FiO_2_ after 72 h of ECMO treatment (all *P* < 0.001) (Fig. 2A). Similarly, LUS and Cdyn also had the same negative correlation (Fig. 2B).

### Receiver operating characteristic analysis

**Fig. 3:**
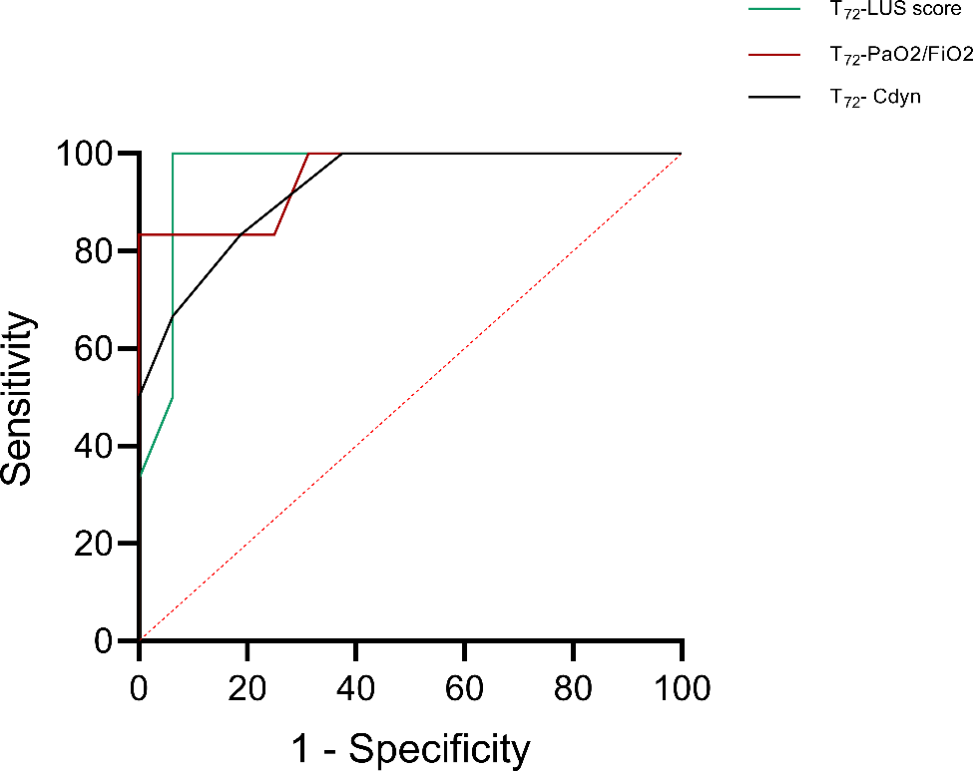
Receiver operating characteristic analysis of the significantly changed variables during ECMO support

Receiver operating characteristic analysis was constructed to determine the diagnostic value of the significantly changed variables during ECMO support for the ICU survival status of the patients. The value of AUC for T_72_-LUS, T_72_-PaO_2_/FiO_2_, T_72_-Cdyn was 0.964[95% *CI*0.887 ~ 1.000], 0.953[95%*CI*0.856 ~ 1.000], 0.927[95%*CI*0.8146 ~ 1.000], respectively (all *P* < 0.05). (Fig. 3 and Table 2).

**Table 2:** Receiver operating characteristic analysis of the significantly changed variables during ECMO support

Parameters	AUC	95% CI	P value	Sensitivity(%)	Specificity(%)
T_72_-LUS score	0.964	0.887 ~ 1.000	*P* < 0.01	100	93.75
T_72_-PaO_2_/FiO_2_	0.953	0.856 ~ 1.000	*P* < 0.01	100	68.75
T_72_-Cdyn	0.927	0.815 ~ 1.000	*P* < 0.01	100	62.50

LUS, lung ultrasound; Cdyn, dynamic lung compliance; ICU, intensive care unit; CI, confidence interval; AUC, area under the curve

## Discussion

Bedside pulmonary ultrasound has been established as an important tool for the evaluation of critically ill patients and has been proven to be helpful in the diagnosis of specific pathological features, such as interstitial oedema, consolidation and pleural effusion [17]. In this retrospective study, dynamic pulmonary ultrasound monitoring was performed on 22 patients who received VA-ECMO treatment for shock. The main findings are as follows: (1) The LUS scores in the nonsurvivor group were higher than those in the survivor group after 72 h of ECMO treatment, and the LUS scores are helpful for assessing the recovery of lung function. (2) T_72_-LUS score is negatively correlated with the value of PaO_2_/FiO_2_.

Lung ultrasonographic examination allows for a rapid and reliable diagnosis of the lung water content and pulmonary ventilation status [18]. As the gold standard for the diagnosis of ARDS, chest CT/X-ray is not suitable for the continuous and rapid pulmonary assessment of patients supported with VA-ECMO due to its transport difficulties and high cost.Hydrostatic pulmonary oedema is one of the serious complications of VA-ECMO support therapy, and increased pulmonary oedema leads to impaired gas exchange, contributing to respiratory failure in ARDS. Systematic prospective multi-institutional studies showed that pulmonary oedema is directly correlated with PaO_2_/FiO_2_ [19]. A strong negative association between the LUS score and Cdyn at 48 h, Day 5 and Day 10 after the commencement of VV-ECMO was observed in adult patients with ARDS [20]. Consistently, our research shows that the LUS scores in the survival group decreased after 72 h of support with VA-ECMO, while PaO_2_/FiO_2_ and Cdyn were the opposite, suggesting that the severity of pulmonary oedema in the survival group was negatively correlated with the duration of VA-ECMO support.

The pathophysiological result of hydrostatic pulmonary oedema caused by VA-ECMO is increased left ventricular (LV) afterload due to reverse ECMO flow in the aorta [3], and patients with a low left stroke volume have a higher risk of this complication [21]. In the survival group, this negative correlation between the LUS score and the duration of VA-ECMO support shows that left ventricular afterload decreased and spontaneous cardiac output increased, which may be related to the recovery of left ventricular systolic function. In the nonsurvivor group, the LUS score increased after 72 h, and PaO_2_/FiO_2_ and Cdyn decreased, suggesting that the LUS score may be an effective reference index for predicting the recovery of left ventricular function in patients with cardiogenic shock, which will be discussed in further research.

Because the PaO_2_/FiO_2_ ratio of VA-ECMO-assisted patients always depends on the ratio between the patient’s spontaneous cardiac output and the ECMO flow and FiO_2_ in the ECMO oxygenator and ventilation setting, oxygenation parameters cannot more accurately indicate the severity of lung function injury in patients during pulmonary oedema episodes [22]. The correlation analysis of the LUS score, PaO_2_/FiO_2_ and Cdyn at various time points during VA-ECMO treatment indicated that the LUS score and PaO_2_/FiO_2_ were significantly correlated after 72 h. This may be related to the blood gas analysis of the right radial artery, which mainly receives oxygenated blood from the patient’s lungs, suggesting that the left ventricular flow gradually increases over time. This once again suggested that the LUS score is an effective and important reference for assessing the severity of lung injury.

An increased incidence of pulmonary oedema during VA-ECMO treatment is closely associated with mortality. Moreover, the LUS score was used to predict the prognosis of ARDS caused by COVID-19 and paediatric diseases, and indicated that the LUS score was associated with a higher risk of PICU mortality and longer PICU stay days after 72 h of VV-ECMO support [23]. Similarly, our study showed that the LUS score in the survival group was significantly lower than that in the nonsurvival group, and the AUC for T_72_-LUS was 0.964 [95% CI 0.887 ~ 1.000]. These findings indicated that lower LUS scores might be linked with better patient survival. Nonetheless, future regression analysis is needed to explore whether LUS scores can function as an independent risk factor or predictor of the survival of patients with cardiogenic shock in the ICU receiving VA-ECMO support. Given the small sample size of the current study, the analysis was not conducted.

There are several limitations of our study. First, it is important to note the retrospective design and the single-centre setting as limitations and that the small sample size may have led to selection bias. For further research, the sample size should be expanded for a stratified analysis. Second, the evaluation of back partitioning requires the patient to be moved, which increases the difficulty of nursing and has the risk of catheter displacement, and multiple measurements are required to ensure the accuracy of the data.Third, left heart decompression procedures have been implemented by numerous medical centers to improve pulmonary edema and lung ultrasound scores. However, it should be noted that our study did not include patients undergoing left heart decompression, therefore, our findings cannot be generalized to this population.

## Conclusions

In conclusion, LUS can be an important means for the dynamic and continuous assessment of lung function in patients with ECMO and can accurately assess the degree of lung lesions in critically ill patients, and the LUS score is expected to be a valuable reference index for predicting ICU prognosis in patients receiving VA-ECMO treatment for cardiogenic shock.

## Data Availability

The datasets generated and analyzed during the current study are not publicly available due to subjects privacy concerns but are available from the corresponding author on reasonable request.

## Abbreviations

LUS: Lung ultrasound
VA-ECMO: Venoarterial extracorporeal membrane oxygenation
Cdyn: Dynamic lung compliance
ICU: Intensive care unit
CI: Confidence interval
CT: Computed tomography
ARDS: Acute respiratory distress syndrome
CRP: C-reactive protein
APACHE II: Acute Physiology and Chronic Health Evaluation II
BMI: Body mass index
PEEP: Expiratory pressure
